# Caution on the Use of ^68^Ga-DOTATATE for the Diagnosis of Pheochromocytoma: A Report of 2 Cases

**DOI:** 10.1210/jcemcr/luad149

**Published:** 2023-12-01

**Authors:** Leor Needleman, Sheila Enamandram, Justin P Annes

**Affiliations:** Department of Medicine, Division of Endocrinology, Stanford University, Stanford, CA 94305, USA; Department of Radiology, Stanford University, Stanford, CA 94305, USA; Department of Medicine, Division of Endocrinology, Stanford University, Stanford, CA 94305, USA

**Keywords:** pheochromocytoma, intra-adrenal sympathetic paraganglioma, ^68^Ga-DOTATATE, RET, SDHC, SDH, SDHx, PET

## Abstract

Pheochromocytomas are intra-adrenal sympathetic neuroendocrine tumors that arise from chromaffin cells. Paragangliomas similarly arise from chromaffin cells, although at extra-adrenal sites such as sympathetic paraganglia in the abdomen/thorax, or parasympathetic paraganglia in the head/neck. Collectively, pheochromocytomas and paragangliomas are important to diagnose and resect because they may secrete harmful levels of catecholamines, have mass effects, hemorrhage, and/or metastasize. Anatomic imaging of pheochromocytomas is usually completed with computed tomography or magnetic resonance imaging; however, functional imaging may be used to provide additional localization, staging, and/or biologic information. Accordingly, selection of the proper functional imaging modality can be critical to developing the optimal therapeutic strategy. ^68^Gallium- and ^64^Copper-1,4,7,10-tetraazacyclododecane-1,4,7,10-tetraacetic acid (DOTA)-octreotate positron emission tomography computed tomography (^68^Ga- and ^64^Cu-DOTATATE) are widely used in evaluating pheochromocytomas and paragangliomas, although data regarding the sensitivity for diagnosing pheochromocytoma are limited. We report 2 cases of pheochromocytoma that showed nondiagnostic ^68^Ga-DOTATATE uptake but were subsequently visualized using alternative functional imaging modalities. Additionally, we provide a review of the literature to highlight the underappreciated limitations of functional adrenal imaging with somatostatin-based compounds.

## Introduction

Pheochromocytoma and paraganglioma (PPGL) represent a subset of neuroendocrine tumors with distinct clinical, biochemical, and anatomic features. PPGL of the adrenal medulla usually secrete excess catecholamines and catecholamine metabolites. Paragangliomas that arise from sympathetic paraganglia in the abdomen or thorax may also overproduce catecholamines, whereas those originating in parasympathetic paraganglia in the head/neck rarely secrete catecholamines. PPGLs are frequently hereditary with an identifiable causative germline mutation, in 1 of more than a dozen genes, evident in approximately one-third of cases in adults.

Anatomic imaging of the adrenal glands (computed tomography [CT] or magnetic resonance imaging [MRI]) is used to diagnose, localize, and characterize pheochromocytomas. Nuclear medicine studies provide additional diagnostic, localization, staging, and biologic information. Notably, the biologic heterogeneity of pheochromocytomas is reflected in the variable sensitivity/specificity of the distinct imaging modalities. Consequently, selecting the appropriate modality for individual patients is essential to providing appropriate diagnostic, staging, and therapeutic guidance. We report 2 cases of pheochromocytoma with nondiagnostic ^68^Gallium-1,4,7,10-tetraazacyclododecane-1,4,7,10-tetraacetic acid (DOTA)-octreotate (^68^Ga-DOTATATE) uptake. Although the sensitivity and specificity of ^68^Ga-DOTATATE for PPGL is widely regarded to be exceptional, most studies focus on metastatic lesion identification rather than primary tumor diagnosis and do not intentionally assess PPGL diagnostic performance (1, 2). Hence, there is limited knowledge and awareness about the diagnostic limitations of ^68^Ga-DOTATATE for pheochromocytoma.

## Case Presentation

### Case 1

An asymptomatic 60-year-old woman was incidentally found to have a pathogenic variant of the *SDHC* gene (c.397C > T (p.Arg133*)). Genetic testing was obtained because her sister was diagnosed with breast cancer and her father was diagnosed with prostate cancer. The patient had no personal or family history of SDHC-related disease, including PPGL, gastrointestinal stromal tumor, or renal cell carcinoma. She was normotensive and physical examination was unremarkable.

### Case 2

An asymptomatic 35-year-old woman was found to harbor a familial (high-risk) pathogenic *RET* gene variant (c.1901G > T (p.Cys634Phe)). Her father died at age 38 years from unspecified complications of pheochromocytoma. Medullary thyroid carcinoma was diagnosed in her paternal grandmother and 3 paternal cousins. The patient's initial evaluation revealed mild diastolic hypertension. Physical examination was negative for palpable thyroid nodules.

## Diagnostic Assessment

### Case 1

**Figure 1.**
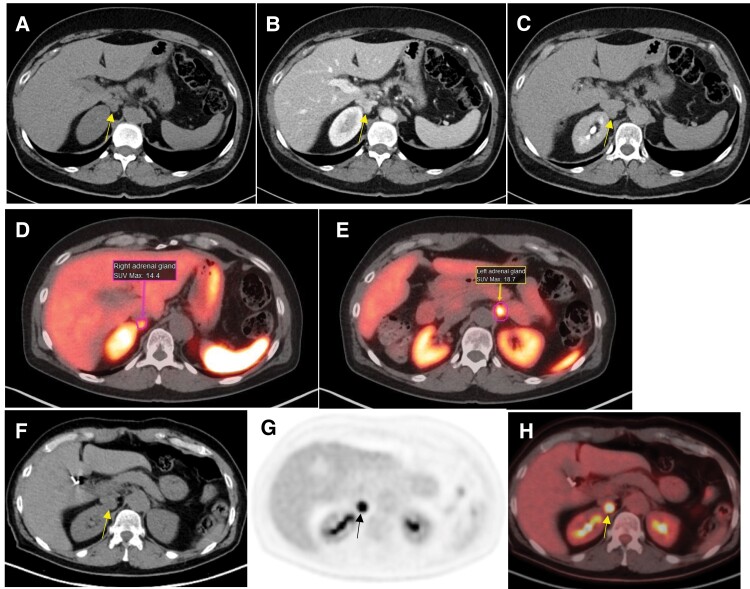
Axial computed tomography (CT) demonstrates a 12 × 9 × 8-mm right adrenal nodule. The right adrenal nodule exhibits an unenhanced CT attenuation of 31 Hounsfield units (HU) (A). The venous phase shows marked enhancement (160 HU) of the adrenal nodule (B). The nodule exhibited 15-minute delayed attenuation of 68 HU; the calculated 71% absolute washout was consistent with a benign lesion (C). ^68^Ga-DOTATATE PET/CT was nondiagnostic for pheochromocytoma, showing a maximum standardized uptake value (SUVmax) of the right adrenal gland of 14.4, less than that of the left adrenal gland SUVmax 18.7 (D and E); this was thus interpreted as physiologic distribution of radiotracer within bilateral adrenal glands. ^18^F-FDG PET/CT demonstrated the right adrenal nodule (F), with ^18^F-FDG axial stand-alone PET image showing intense right adrenal uptake (G). The fused ^18^F-FDG PET/CT image confirmed the right adrenal nodule was highly avid, with SUVmax 23.2 (H), compatible with a right adrenal pheochromocytoma.

Measurement of plasma metanephrines showed marginally elevated normetanephrine (219 pg/mL [1.2 nmol/L], reference range <148 pg/mL), whereas 24-hour urine metanephrines were normal. An adrenal CT scan revealed an enhancing 1.2-cm right adrenal nodule with an unenhanced CT attenuation of 31 Hounsfield units (HU) (Fig. 1A). Fifteen minutes after administration, there was an absolute 71% contrast washout (Fig. 1B and C). Based on the pathogenic *SDHC* gene mutation, ^68^Ga-DOTATATE PET/CT was performed to confirm a suspected pheochromocytoma and assess for paragangliomas. The ^68^Ga-DOTATATE positron emission tomography (PET)/CT demonstrated bilateral adrenal positivity (Fig. 1D and E), interpreted as “physiologic.” Subsequently, ^18^F-fluorodeoxyglucose (^18^F-FDG) PET/CT, obtained to clarify the diagnosis, demonstrated intense uptake in the right adrenal nodule (Fig. 1F and G, H). No additional FDG-avid lesions were present.

### Case 2

**Figure 2.**
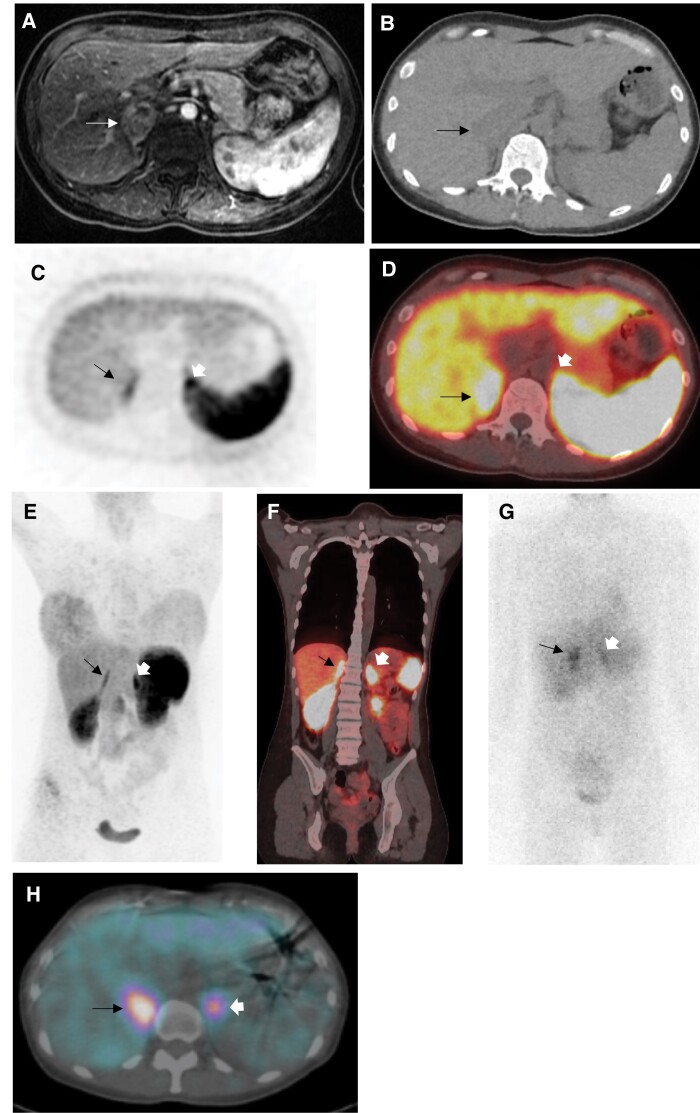
T1-weighted postcontrast axial magnetic resonance imaging (MRI) showing a 2.7 × 2.7 × 4.1-cm enhancing mass in the right adrenal gland, with punctate hyperintensity in the periphery of the lesion likely reflecting calcifications or blood products (A). ^68^Ga-DOTATATE PET/CT failed to demonstrate avidity within the right adrenal mass. A CT image from the ^68^Ga-DOTATATE PET/CT study shows the right adrenal mass (B). Axial standalone PET and fused ^68^Ga-DOTATATE PET/CT images were interpreted as showing physiologic uptake in both adrenal glands (right adrenal = arrow, left adrenal = arrowhead) (C and D). The ^68^Ga-DOTATATE PET maximum intensity projection (MIP) whole-body image demonstrated similar findings when compared with axial (E). Measurement of SUVmax on the fused coronal ^68^Ga-DOTATATE PET/CT shows that the right adrenal gland (SUVmax 9.7) had less DOTATATE uptake than the left adrenal gland (SUVmax 15.5) (F). ^123^I-MIBG imaging showed significantly increased uptake in the right adrenal mass consistent with pheochromocytoma. The right adrenal gland had greater ^123^I-MIBG uptake than the left adrenal gland on the anterior whole body planar image (G). The fused axial ^123^I-MIBG SPECT/CT shows focally and asymmetrically intense uptake in the right adrenal mass compatible with pheochromocytoma (H). The relatively high ^123^I-MIBG uptake in the left adrenal gland was interpreted as physiologic.

At diagnosis, screening for hyperparathyroidism was normal with serum calcium of 9.3 mg/dL (2.33 mmol/L; reference range, 8.4-10.5 mg/dL) and PTH of 23 pg/mL (reference range, 15-65 pg/mL). Screening for medullary thyroid cancer showed elevated serum calcitonin of 42 pg/mL (12.26 pmol/L; reference range, <7.6 pg/mL). Plasma metanephrines were also abnormal, with elevated normetanephrine (5.0 nmol/L [916 pg/mL]; reference range, <.90 nmol/L) and metanephrine (1.6 nmol/L [304 pg/mL]; reference range, <.50 nmol/L). Thyroid ultrasound showed two .2-cm solid hypoechoic nodules (both TI-RADS 4). MRI of the abdomen revealed an enhancing 4.1-cm right adrenal mass without signal loss on out-of-phase imaging (Fig. 2A). Two nodules (5 and 8 mm) were also noted in the left adrenal gland. ^68^Ga-DOTATATE PET/CT, performed to assess possible bilateral pheochromocytomas and metastasis, showed physiologic uptake bilaterally (Fig. 2B-F). ^123^I-metaiodobenzylguanidine scintigraphy (^123^I-MIBG) was obtained to resolve whether bilateral disease was present; increased signal was observed in the right adrenal mass only (Fig. 2G and H).

## Treatment

### Case 1

Preoperative blockade was deferred in favor of close intraoperative hemodynamic monitoring at the anesthesiologist's request; the patient was asymptomatic, home blood pressure monitoring was normal, only mild catecholamine excess was present, and the tumor was of small size. She underwent an uneventful laparoscopic total right adrenalectomy.

### Case 2

The patient was started on α-adrenergic blockade with doxazosin 1 mg daily, which was up-titrated to 2 mg daily. She underwent laparoscopic cortical-sparing right adrenalectomy followed by total thyroidectomy. She tolerated surgery well and doxazosin was discontinued with resolution of diastolic hypertension.

## Outcome and Follow-up

### Case 1

The patient had complete recovery. Pathology revealed a 1.4-cm pheochromocytoma with loss of SDHB immunostaining. There was no invasion through the adrenal capsule. At less than 1 year since surgery, continued biochemical and radiologic tumor surveillance remain negative.

### Case 2

Adrenal pathology showed a 3.8-cm pheochromocytoma with focal capsular invasion, without involvement of periadrenal fat. Thyroid pathology showed .2-cm and .1-cm medullary thyroid microcarcinomas. Postoperatively, the patient initiated thyroid replacement and has maintained normal plasma metanephrines and calcitonin levels (5 years). Surveillance MRI scans demonstrate stable left adrenal thickening without evidence for residual/recurrent right adrenal disease.

## Discussion

We report 2 cases of hereditary pheochromocytoma with false-negative ^68^Ga-DOTATATE PET imaging. The first case describes a patient with hereditary pheochromocytoma paraganglioma syndrome, caused by a pathogenic loss-of-function mutation of *SDHC*, which demonstrates autosomal dominant inheritance with incomplete penetrance. The mitochondrial SDH enzyme catalyzes the conversion of succinate to fumarate in the tricarboxylic acid cycle and functions as complex II of the electron transport chain. SDH-deficient tumors accumulate succinate, which acts as an oncometabolite that contributes to tumorigenesis (3). Case 2 also describes a patient with a familial tumor syndrome, multiple endocrine neoplasia type 2A (MEN2A). MEN2A is characterized by the development of medullary thyroid carcinoma, pheochromocytoma, and primary hyperparathyroidism and is inherited in an autosomal dominant manner. MEN2A is caused by germline gain-of-function mutations in the *RET* proto-oncogene, which encodes a receptor tyrosine kinase. Most MEN2A cases result from mutation within the extracellular cysteine-rich domain of RET, resulting in ligand-independent receptor dimerization, and constitutive cell growth signaling. Pheochromocytomas associated with mutated tricarboxylic acid cycle enzymes are typically normetanephrine producing, without substantial metanephrine elevation, whereas RET mutation-associated pheochromocytomas typically exhibit a mixed biochemical phenotype (elevations of both normetanephrine and metanephrine).

The diagnosis and appropriate treatment of pheochromocytomas is essential because of the risks of acute and chronic exposure to catecholamine excess, the presence of malignant potential, and the potential to identify causative germline mutations that allow early diagnosis in at-risk family members. Proper management requires tumor localization, medical management of functional tumors, and surgical resection when practical. Because selection of the optimal surgical approach depends on reliable assessment of the extent of disease, it is important for clinicians to recognize the limitations of each imaging modality. Imaging with radiolabeled somatostatin analogs has demonstrated utility for characterizing neuroendocrine tumors, including pheochromocytomas, which exhibit increased somatostatin receptor expression. ^68^Ga-DOTATATE is a radiolabeled somatostatin analog that has demonstrated superiority for detection of PPGL at extra-adrenal sites. ^64^Cu-DOTATATE (expected to have similar performance to ^68^Ga-DOTATATE) is increasingly used by imaging centers because its longer half-life makes it easier to use in the clinical setting. In contrast,^123^ I-MIBG, which relies on the structural similarity of iobenguane to norepinephrine, may be used to identify neuroendocrine tumors with enhanced catecholamine storage but has lower detection rates than ^68^Ga-DOTATATE for paragangliomas and metastatic PPGL. ^18^F-fluorodihydroxyphenylalanine (^18^F-FDOPA) is a radiolabeled amino acid that takes advantage of upregulated amino acid transport by neuroendocrine tumors and has relatively high sensitivity for PPGL but is not widely available. ^18^F-FDG is useful for patients with primary and metastatic PPGL, although it lacks specificity for neuroendocrine tumors.

The choice of functional imaging study is influenced by characteristics of each case, radiotherapy planning, and radiopharmaceutical availability. Clinical practice guidelines for PPGL recommend a personalized approach for tumor localization with functional imaging (4). Although MRI identified the right pheochromocytoma in case 2, ^68^Ga-DOTATATE PET/CT was used to determine whether bilateral pheochromocytomas were present and did not demonstrate increased right adrenal uptake. Although failure to visualize the MEN2A-associated right pheochromocytoma with ^68^Ga-DOTATATE was unexpected, previous work has shown a higher detection rate in MEN2A-related pheochromocytoma patients using anatomic imaging (5). In addition, the greater ^68^Ga-DOTATATE uptake observed in the patient's left adrenal gland may reflect medullary hyperplasia. Although the sensitivity of somatostatin analog-based imaging for adrenal pheochromocytoma is excellent, it is not perfect, and clinicians should be aware of this limitation. In a prospective comparison of ^68^Ga-DOTATATE and ^18^F-FDOPA, the sensitivity of ^68^Ga-DOTATATE was higher for parasympathetic paraganglia in the head/neck (HNPGL) (100%) but lower for pheochromocytoma (73%) (2). Greater sensitivity for HNPGL was affirmed by a subsequent metanalysis of 9 studies of PPGL detection with ^68^Ga-DOTA-conjugated somatostatin receptor-targeting peptides (^68^Ga-DOTA-SST) (6). Higher detection rates with ^68^Ga-DOTA-SST were reported in studies that had a greater prevalence of HNPGL. One explanation for lower sensitivity for pheochromocytoma is that ^68^Ga-DOTATATE is taken up by healthy adrenal glands. In a study of preoperative assessment of PPGL cases using ^68^Ga-DOTATATE, the mean maximum standardized uptake value (SUVmax) was not significantly different between confirmed pheochromocytomas and normal adrenal glands (7). The mean SUVmax of PGLs at extra-adrenal sites was significantly higher than normal adrenals, and the sensitivity of ^68^Ga-DOTATATE was 88% for pheochromocytoma vs 100% for PGL. Consequently, ^68^Ga-DOTATATE may have reduced sensitivity for smaller pheochromocytomas, consistent with case 1 of the present study. False-negative ^68^Ga-DOTATATE imaging of a small pheochromocytoma within a highly avid adrenal gland is also described in the literature (2).

Another potential cause of low ^68^Ga-DOTATATE sensitivity for pheochromocytoma is reduced somatostatin receptor (SSTR) expression. ^68^Ga-DOTA-SST uptake depends on the tumor expression profile of SSTR subtypes. Because ^68^Ga-DOTATATE is most specific for SSTR2 and SSTR5, lower sensitivity for pheochromocytoma may be caused by absence of these SSTR subtypes on some tumors. Studies of SSTR expression in pheochromocytoma have yielded variable results. In 1 study of immunostaining for SSTR subtypes in 52 pheochromocytomas, SSTR3 was positive in 90% of tumors, whereas only 25% and 15% were positive for SSTR2 and SSTR5, respectively (8). A recent analysis of patients with PPGL showed that for 202 tumor samples, 50% had positive SSTR2 immunostaining (9). Given these findings, SSTR2 expression on pheochromocytomas likely varies more than expected. A few PPGL cases with high ^18^F-FDG but low ^68^Ga-DOTATATE uptake have been reported in association with aggressive disease, which may reflect loss of SSTR expression resulting from dedifferentiation or necrosis (1).

Although imaging with ^68^Ga-DOTA-SST has proven high PPGL lesion-based detection rates, the sensitivity of ^68^Ga-DOTATATE for pheochromocytoma remains unresolved because most studies have addressed performance at extra-adrenal sites. Several studies of ^68^Ga-DOTATATE performance cited in this report detail their detection rates for pheochromocytoma, and on pooling the data, we estimate a sensitivity of 88% (65/74) (2, 7, 10). Although ^68^Ga-DOTATATE PET/CT maintains important clinical utility for PPGL localization, sensitivity should be considered highest for metastatic PPGL and HNPGL. Clinicians should be aware of the potential for lower sensitivity of ^68^Ga-DOTATATE for pheochromocytoma.

## Learning Points

- A personalized approach is appropriate when choosing functional imaging studies for pheochromocytomas and paragangliomas.
- ^68^Gallium-tetraazacyclododecane-1,4,7,10-tetraacetic acid-octreotate (^68^Ga-DOTATATE) positron emission tomography/computed tomography is a powerful tool for neuroendocrine tumor localization but more studies assessing its sensitivity for pheochromocytoma are needed.
- Pheochromocytoma and paraganglioma detection with ^68^Ga-DOTATATE may be better for metastatic disease and parasympathetic paraganglia in the head/neck.
- False-negative results with ^68^Ga-DOTATATE positron emission tomography/computed tomography for pheochromocytoma may be explained by low SSTR2 expression, tumor necrosis, or high uptake by healthy adrenal glands.
- Additional functional imaging modalities may be necessary for patients with negative ^68^Ga-DOTATATE imaging and high suspicion of having pheochromocytoma.

## Contributors

J.P.A. was involved in the diagnosis and patient management, writing of the manuscript, and manuscript submission. L.N. wrote the manuscript. S.E. provided images used for the figures. All authors reviewed and approved the final draft.

## Data Availability

Data sharing is not applicable to this article as no datasets were generated or analyzed during the current study.

## Abbreviations

^18^F-FDG: ^18^F-fluorodeoxyglucose
^68^Ga-DOTATATE: ^68^Gallium-1,4,7,10-tetraazacyclododecane-1,4,7,10-tetraacetic acid
^123^I-MIBG: ^123^I-metaiodobenzylguanidine scintigraphy
CT: computed tomography
HNPGL: parasympathetic paraganglia in the head/neck
HU: Hounsfield unit
MEN2A: multiple endocrine neoplasia type 2A
MRI: magnetic resonance imaging
PET: positron emission tomography
PPGL: pheochromocytomas and paragangliomas
SSTR: somatostatin receptor
SUVmax: maximum standardized uptake value
